# Effects of soy isoflavone on hepatic steatosis in high fat-induced rats

**DOI:** 10.3164/jcbn.16-98

**Published:** 2017-07-20

**Authors:** Huanhuan Liu, Huijia Zhong, Liang Leng, Zhuoqin Jiang

**Affiliations:** 1 Department of Nutrition, School of Public Health, Sun Yat-Sen University, Guangzhou, 510080, China

**Keywords:** soy isoflavone, steatosis, SREBP-1c, PPARα, NAFLD

## Abstract

Soy isoflavone has benefits for metabolic syndrome but the mechanism is not completely understood. This study was designed to determine the effects of soy isoflavone on hepatic fat accumulation in non-alcoholic fatty liver disease (NAFLD) rats induced by high fat diet (HFD). Sprague-Dawley rats were administrated with a normal fat diet (control), HFD (NAFLD model), HFD with 10 or 20 mg/kg soy isoflavone daily for 12 weeks. Hepatic and serum lipid contents, liver histopathological examination, serum alanine transaminase (ALT), protein and mRNA expression of sterol regulatory element binding protein (SREBP)-1c, fatty acid synthase (FAS), peroxisome proliferator-activated receptor (PPAR) α were assayed respectively. Our study found that soy isoflavone reduced HFD-induced lipid accumulation in liver, serum ALT and improved liver lobule structure. In addition, the expression of SREBP-1c and FAS was lower, whereas protein level of PPARα was higher in two soy isoflavone groups than that of the HFD group. Collectively, these results demonstrate that soy isoflavone is capable of alleviating hepatic steatosis and delaying the progression of NAFLD via inhibiting lipogenesis and promoting fatty acid oxidation in liver.

## Introduction

Non-alcoholic fatty liver disease (NAFLD), a chronic progressive disease, may progress from simple steatosis to severe liver damage, and the typical features are intracellular accumulation of triglyceride (TG) and excessive hepatic lipid droplets.^(1–3)^ The pathogeny of NAFLD has not been elucidated clearly, the most widely accepted explanation is “two hit theory”, in which the accumulation of lipids in liver is considered the first hit, the second hit is characterized by oxidative stress and lipid peroxidation on the basis of the first hit, and may contribute to hepatocyte injury.^(4)^ A lot of evidence shows NAFLD could affect multiple systems including extra-hepatic organs and regulatory pathways, and be a main reason of liver-related morbidity and mortality.^(5,6)^ However, the effective drug to treat NAFLD is rather limited today.^(7,8)^

Soy isoflavone, the major soy phytoestrogen, mainly consists of genistein, daidzein and glycitein. Much attention has been paid to its abilities of preventing osteoporosis, cancer, cardiovascular disease and relieving menopausal syndrome.^(9–12)^ Jha *et al.*^(13)^ found soy isoflavone could inhibit microsomal lipid peroxidation. Similarly, Zhao *et al.*^(14)^ observed the ability of isoflavone to lower malondialdehyde in injured liver. Besides, soy isoflavone has hypolipidemic effect and capable of increasing insulin sensitivity, which play important roles in the development of NAFLD.^(15,16)^ The protective mechanism of soy isoflavone on liver may be correlated with the anti-oxidative, hypolipidemic effect and improvement of insulin resistance.

Based on the published reports, soy isoflavone may have beneficial effect on NAFLD rats but the mechanism is not clearly. Sterol regulatory element binding protein (SREBP)-1c is a member of transcription factors and regulates the expression of its down-stream genes, including fatty acid synthase (FAS), acetyl-CoA carboxylase (ACC), thus promoting fat synthesis in liver. Peroxisome proliferator-activated receptor (PPAR) α, highly expressed in liver, is closely related with fatty acid β-oxidation and knockout of hepatocyte PPARα gene could cause hepatic steatosis.^(17)^ In this study, we investigated the effects of long-term consumption of soy isoflavone on hepatic steatosis induced by high fat diet (HFD), and chose SREBP-1c and and PPARα pathways as the targets to investigate the possible mechanisms.

## Materials and Methods

### Animals and treatment

Male Sprague-Dawley rats were obtained from the Experimental Animal Center of Guangdong Province, China. Rats (average body weight 205.20 g) were housed under controlled conditions respectively: temperature kept at 22–26°C and relative humidity at 68–80%, with a 12-h light/dark cycle. After adaptive feeding, these rats were randomly divided into 4 groups (*n* = 9) as follows: i) group 1: the group administrated with D12450B (10% fat energy; control); ii) group 2: the group administrated with D12492 (60% fat energy; HFD); iii) group 3: the group fed with HFD and 10 mg/kg/day soy isoflavone intragastrically (HFD + ISF 10 mg/kg); iv) group 4: the group received HFD and 20 mg/kg/day soy isoflavone intragastrically (HFD + ISF 20 mg/kg). Soy isoflavone was dissolved in distilled water in a volume of 0.5 ml/100 g, while both the control and HFD rats were treated with the same volumes of vehicle solution only. Based on the previous literature published, we made some minor modification and succeeded to duplicate the rat model of NAFLD by HFD.^(18)^ The study was conducted for 12 weeks, all rats allowed water *ad libitum*, and rats got HFD were accompanied by 18% saccharose solution freely. Weight was measured weekly. Soy isoflavone (purity, 90.04%, bought from North China Pharmaceutical Group Co., Ltd., China) contains 28.52% genistin, 1.24% genistein, 40.59% daidzin, 0.78% daidzein, 18.84% glycitin and 0.07% glycitein. The project was approved by the animal experimental ethics committee of Sun Yat-Sen University, China.

### Samples collection

12 weeks later, the rats were fasted overnight. Under pentobarbital anesthesia, 4–5 ml blood was withdrawn from the abdominal aorta rapidly. Subsequently the serum was obtained after the blood was clotting (30 min, 25°C) and centrifuged (2,000 × *g*, 10 min, 4°C), then saved at −80°C until analysis. After sacrifice, liver was rapidly separated and weighted, then immediately frozen at −80°C or fixed in 10% formaldehyde for the subsequent experiments.

### Measurement of ALT and lipid profiles in liver and serum

ALT, TG and total cholesterol (TC) were measured by the appropriate kits (Roche, China; Biosino, China) respectively on fully automatic biochemical analyzer (Hitachi 917, Tokyo, Japan). Free fatty acid (FFA) was assayed by the kit (Cayman, Ann Arbor, MI) according to enzyme-linked immunosorbent assay (ELISA).

### Hematoxylin and eosin (H&E) and Oil-Red O staining

For histopathological examination, Paraffin-embedded liver was sliced and stained with H&E, then deparaffinized in xylene and hydrated in graded ethanol. To demonstrate fat accumulation in liver, hepatic tissues were sliced in 8 µm thickness with a freezing microtome (Leica, Wetzlar, Germany). After rinsing with stilled water and staining with Oil-Red O (Sigma, St. Louis, MO) for 10 min, fat accumulation in the obtained liver preparations was observed under light microscope (Nikon, Tokyo, Japan).

### Western blotting

Proteins of liver SREBP-1c, PPARα and FAS were determined by western blotting according to the commercially available kits (Key GEN, China) under the standard procedure. The protein contents of the extracts were determined by bicinchoninic acid (BCA) assay (Beyotime, China). 20 µg protein was loaded and separated through SDS-PAGE and transferred onto PVDF (Millipore, Boston, MA). After keeping with 5% skim milk for 1 h, the rabbit polyclonal antibodies against SREBP-1c, PPARα, FAS, Histone 2H.X (1:1,000; Santa Cruz, Santa Cruz, CA) and monoclonal antibody against GAPDH (1:2,000; Santa Cruz) were added and the membranes were subsequently incubated overnight at 4°C; Subsequently, incubated with HRP-conjugated secondary antibody (1:5,000) for 1 h. The relative SREBP-1c, FAS and PPARα protein-levels were analyzed using the Quantity one.

### Real-time RT-PCR

Total RNA in liver tissue was separated by Trizol reagent (Invitrogen, Carlsbad, CA) under the manufacturer’s direction. Subsequently reverse transcription of total RNA (5 µg) was conducted by the commercial kit (Fermentas, Burlington, Ontario). The primer sequences for rat SREBP-1c, PPARα, and β-actin were depicted in Table 1. After reverse transcription, PCR was conducted using SYBR-Green Master Mix kit (Tiangen Biochemical Technology Co., Ltd., China). The specificity of PCR products were performed by a melting curve analysis, and changes in fluorescence during the PCR were calculated under the supplier’s instruction. The relative amount of a target gene was normalized by β-actin, just as previously described.^(19)^ The relative expression ratio (R) was obtained according to the following equation: R = 2 − ΔΔCt.^(20)^

### Statistical analysis

Results are presented as means ± SD, and analyzed using SPSS 13.0. One-way ANOVA or Games-Howel were performed depending on data normality with *p*<0.05 indicating significant differences.

## Results

### Food intake, liver weight, body weight and liver index

Food intake of HFD rats was less than that of the control group, but soy isoflavone administration didn’t change food consumption compared with HFD rats (data not shown). In HFD group, liver weight, body weight and liver index were all elevated, especially the liver weight and liver index of HFD rats were nearly as twice as that of the control group. The supplement of soy isoflavone could significantly reduce liver weight and liver index (*p*<0.01), but not body weight (*p*>0.05) (Table 2).

### Macroscopic results of rat liver

**Fig. 1:**
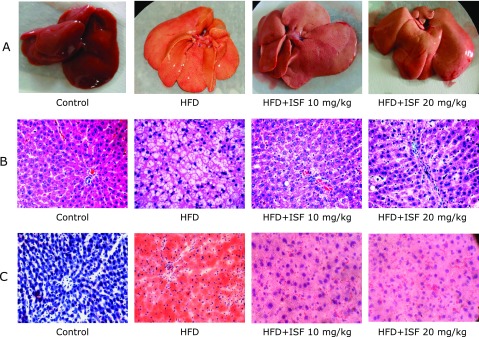
Soy isoflavone improves hepatic steatosis macroscopicly and histologically. Liver sample was separated from the rats at the end of the 12th week. Then (A) macroscopic changes and histological analysis of liver tissues with (B) H&E staining (400×), and (C) Oil-Red O staining (400×) were observed under light microscope.

Rat liver in the control group was dark red, soft, sharp-edged, section smooth. Whereas HFD rat liver was larger, obtuse, and greasy. The color and texture of livers in high and low soy isoflavone dose group were improved compared with HFD group (Fig. 1A).

### Microscopic histopathologic results of rat liver

Liver sections were stained with H&E. No microscopic steatosis was detected in the hepatocyte of the control group rats, and nuclei of hepatocytes were of normal appearance and location. However, hepatocyte from HFD rats was enlarged and filled with lipid droplets. Meanwhile disorganized structure of hepatic cord and nuclear translocation were also observed. Hepatic steatosis was dramatically improved in both soy isoflavone groups, especially the high-dose soy isoflavone administration (Fig. 1B). For the better observation of fat deposition in liver, the liver specimens were stained with Oil-Red O. Results were similar to the H&E staining and depicted more clearly (Fig. 1C).

### ALT in serum

Serum ALT content in HFD rats was much higher than that of the control group (*p*<0.05). Both high- and low-dose soy isoflavone supplementation could reduce ALT content to nearly normal values (Table 2).

### Lipid profiles in liver and serum

**Fig. 2:**
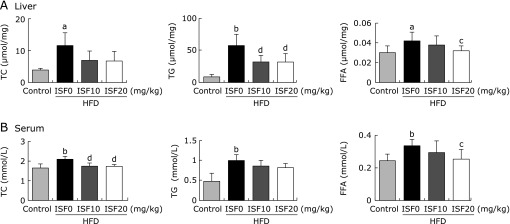
Soy isoflavone regulates total cholesterol (TC), triglyceride (TG) and free fatty acid (FFA) in (A) liver and (B) serum. Values are the mean ± SD, *n* = 8 or 9. ^a^*p*<0.05, ^b^*p*<0.01 compared to the control; ^c^*p*<0.05, ^d^*p*<0.01 compared to the HFD group.

Concentration of TC, TG and FFA in liver and serum from the HFD rats was increased (*p*<0.05); In liver, however, the content of hepatic FFA in high-dose soy isoflavone rats was decreased (*p*<0.05); Soy isoflavone administration in the two groups significantly decreased the contents of hepatic TG compared with the HFD group (*p*<0.01); Similarly, in serum, low- or high-dose soy isoflavone treatment could decrease the concentration of TC and FFA compared with that of the HFD rats (*p*<0.01, *p*<0.05), especially the high-dose soy isoflavone was able to restore the level of FFA to nearly normal. There wasn’t difference between the two soy isoflavone groups (Fig. 2).

### Protein expression of SREBP-1c, FAS and PPARα in liver

**Fig. 3:**
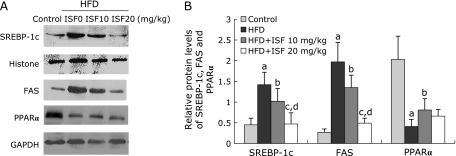
Soy isoflavone affects protein levels of sterol regulatory element binding protein (SREBP)-1c, fatty acid synthase (FAS) and peroxisome proliferator-activated receptor (PPAR) α in liver. (A) The content of protein expression was shown by western blotting images. (B) The relative expression level was normalized to Histone or GAPDH protein. Values are the mean ± SD, *n* = 8 or 9. ^a^*p*<0.01 compared to the control group; ^b^*p*<0.05, ^c^*p*<0.01 compared to the HFD group; ^d^*p*<0.01 compared to the low-dose soy isoflavone group.

The level of SREBP-1c and FAS protein expression in liver from the HFD rats was much higher than the control (*p*<0.01). However, soy isoflavone showed obvious restorations on the protein expression of SREBP-1c and FAS in a dose dependent manner. HFD dramatically decreased the hepatic protein expression of PPARα (*p*<0.01). The outcome could be reversed by low dose soy isoflavone treatment (*p*<0.01), but not high dose soy isoflavone diet (Fig. 3A and B).

### mRNA expression of SREBP-1c and PPARα in liver

**Fig. 4:**
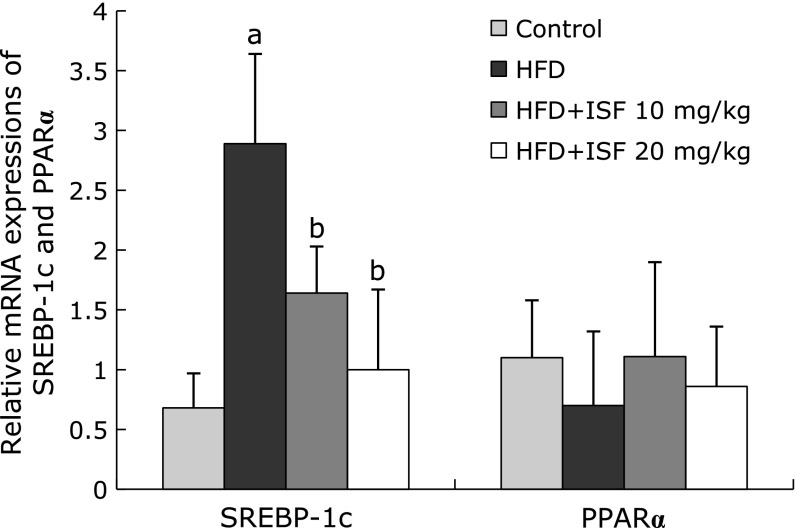
Soy isoflanove regulates mRNA expression of sterol regulatory element binding protein (SREBP)-1c and peroxisome proliferator-activated receptor (PPAR) α in liver. The liver samples were obtained 12 weeks later, the mRNA level was determined by real-time RT-PCR, and was normalized to β-actin. Values are the mean ± SD, *n* = 8 or 9. ^a^*p*<0.01 compared to the control; ^b^*p*<0.01 compared to the HFD group.

 The expression level of SREBP-1c in HFD rats was markedly elevated compared with that of the control group (*p*<0.01). The result was significantly counteracted by the administration of soy isoflavone (*p*<0.01). As for the level of PPARα mRNA, however, no differences exist among the four groups (*p*>0.05) (Fig. 4).

## Discussion

Our result showed that soy isoflavone could reduce fat deposits in liver via the following mechanism: (1) lowers SREBP-1c- and FAS-mediated lipogenesis; (2) activates the expression of PPARα to promote fatty acid oxidation in liver.

In our study, we successfully reproduced the model of NAFLD, of which the typical features was histologically hepatocellular lipid deposition. ALT is an indicator of liver function to determine whether the liver is normal. In this study, we also observed increased body weight, liver weight, blood lipids and serum ALT similar to the previous investigation.^(21,22)^ As expected, soy isoflavone could slow down the progression of NAFLD by reducing liver index, ALT and improving liver structure histopathologically but not changing food consumption.

Hepatic steatosis, as the first hit of the “two hit theory”, is induced by the increasing sources and decreasing output. The patients with NAFLD usually accompany with higher TG, TC, LDL and lower HDL,^(23,24)^ thus much lipid are absorbed to liver. Another source of hepatic lipid is de novo lipogenesis (DNL), during which many rate-limiting enzyme exist, including FAS and ACC. TG in liver would be oxidized or transported to extracellular by VLDL,^(25)^ once the balance of source and output is broken, TG will accumulate in liver. In our study, the levels of hepatic and serum TG, TC, and FFA in NAFLD rats were much higher than that of the normal group, but soy isoflavone significantly decreased not only FFA and TG in liver, but also TC and FFA in serum, this was consistent with the previous investigation.^(26)^

SREBP-1c can bind to the promoters regions of its target genes, and then promote the transcript of lipogenic enzyme; interestingly SREBP-1c can also combine with its own promoter and activate the expression of itself; in addition, it can be activated by liver X receptor (LXR).^(27)^ In NAFLD, hepatic expression of SREBP1-c and FAS were elevated, this may be a causative reason for the increasing of lipid production in liver. While in our study the level of SREBP-1c protein and mRNA were down-regulated by soy isoflavone especially in the high-dose group, the same as the protein expression of FAS. This was in accordance with the previous studies that soy isoflavone was able to suppress the expression of SREBP-1c and FAS *in vitro* and *in vivo*.^(28,29)^ Whether the inhibition of SREBP-1c expression by soy isoflavone is mediated by LXR or other regulatory factors, further studies are needed to conduct.

PPARα is a member of nuclear receptor superfamily. Once activated, PPARα would bind to the DNA-bonding site of the down-stream genes in the form of PPAR-RXR heterodimer, thus regulate key fatty acid oxidation enzymes, such as ACOX1, CPT-I, CPT-II, etc.^(30)^ The synthetic PPARα agonists are widely used in clinical to lower plasma lipid and improve fatty liver.^(31)^ While ablation of PPARα gene could increase susceptibility to NAFLD in high fat-induced rats.^(32)^ As shown in our study, the level of PPARα was lower in NAFLD rat liver, while soy isoflavone increased the protein expression of PPARα. The result was in accordance with the previous studies.^(26,33,34)^ There was evidence show that genistein (the major components of soy isoflavone) may act as a natural agonist of PPARγ (another isotype of PPAR superfamily).^(35)^ Accordingly, we would speculate that certain components of soy isoflavone, such as genistein and daidzein, may act as a ligand of PPARα, thus activate the target genes about fatty acid oxidation. More studies are needed to validate the hypothesis.

In summary, soy isoflavone inhibits the synthesis of fatty acid and promote fat oxidation in NAFLD rat liver by regulating the expression of SREBP-1c and PPARα. However, many unanswered questions still exist: how soy isoflavone actives SREBP-1c and PPARα, whether there are other potential signal transduction pathways to regulate lipid metabolism and so on. Further investigations are essential to clarify the potentially beneficial effects and specific molecular mechanisms of soy isoflavone on NAFLD.

## Figures and Tables

**Table 1:** Primer sequences used for real-time RT-PCR

Primer	Sequences (5'-3')	Amplicon length (bp)
SREBP-1c	GCTGCTCCTGTGTGATCTACTTCT CTGGGGTCCATTGCTGGTA	106
PPARα	ATGCCCTCGAACTGGATGAC CAATCCCCTCCTGCAACTTC	117
β-actin	ATGTGGCCGAGGACTTTGAT TGGCTTTTAGGATGGCAAGG	100

**Table 2:** Body weight, liver weight, liver index and ALT in different groups

	Initial body weight (g)	Final body weight (g)	Liver weight (g)	Liver index (%)	ALT (U/L)
Control	202.91 ± 6.93	481.83 ± 30.14	11.31 ± 0.95	2.34 ± 0.07	22.13 ± 3.40
HFD	199.98 ± 13.64	535.68 ± 33.00^a^	20.85 ± 1.89^a^	3.91 ± 0.41^a^	39.33 ± 8.94^a^
HFD + ISF 10 mg/kg	208.24 ± 7.14	508.96 ± 27.65	16.84 ± 0.92^c^	3.32 ± 0.32^c^	29.00 ± 3.71^b^
HFD + ISF 20 mg/kg	208.93 ± 8.44	508.79 ± 25.04	16.33 ± 1.38^c^	3.32 ± 0.31^c^	26.38 ± 4.17^c^

Values are the mean ± SD, *n* = 8 or 9. ^a^*p*<0.01 compared to the control group; ^b^*p*<0.05, ^c^*p*<0.01 compared to the HFD group. The liver index was calculated as liver weight/body weight × 100.

## Abbreviations

ACC: acetyl-CoA carboxylase
ALT: alanine transaminase
DNL: *de novo* lipogenesis
ELISA: enzyme-linked immunosorbent assay
FAS: fatty acid synthase
FFA: free fatty acid
H&E: hematoxylin and eosin
HFD: high fat diet
LXR: liver X receptor
NAFLD: non-alcoholic fatty liver disease
PPAR: peroxisome proliferator-activated receptor
RT-PCR: reverse transcription polymerase chain reaction
SREBP-1c: sterol regulatory element binding protein-1c
TC: total cholesterol
TG: triglyceride
